# Panscleritis masquerading as an attack of primary acute angle closure glaucoma

**DOI:** 10.3205/oc000120

**Published:** 2019-08-20

**Authors:** Hafsa Bashir, Uma Sridhar, Shahana Mazumdar, Koushik Tripathy

**Affiliations:** 1 Department of Ophthalmology, ICARE Eye Hospital & Postgraduate Institute, Noida, India

**Keywords:** congestive glaucoma, secondary glaucoma, anterior rotation of ciliary body, pupillary block glaucoma

## Abstract

**Purpose:** To report a female who presented with acute angle closure glaucoma and was found to have panscleritis on further evaluation.

**Method:** Case report.

**Case description:** A 50-year-old female was referred to us as a case of primary acute angle closure attack in the right eye and for laser peripheral iridotomy. She had severe pain, redness, a very shallow anterior chamber, and an intraocular pressure of 38 mmHg in the right eye. However, the fellow eye had a deep anterior chamber and the right eye also had severe chemosis, lid edema, scleral tenderness, choroidal folds, and pain during ocular movements which was limited. Ultrasound biomicroscopy showed a ciliochoroidal effusion with anterior rotation of the ciliary body. The ultrasound of the eye revealed an increased thickness of the ocular coats and subtenon fluid. A diagnosis of panscleritis causing secondary angle closure glaucoma was made. She responded well to topical atropine, and topical with systemic steroids.

**Conclusions:** Secondary angle closure glaucoma due to panscleritis may mimic primary acute angle closure attack in a clinical setting. It is important to differentiate the two as treatment is opposite and may worsen the condition if misdiagnosed.

## Introduction

Primary angle closure glaucoma (ACG) attack is an ophthalmic emergency mediated by pupillary block. Immediate reduction of the intraocular pressure is of utmost importance to preserve visual acuity. The management includes reduction of the intraocular pressure using topical and systemic antiglaucoma medications, pupillary constriction using pilocarpine, and laser peripheral iridotomy (PI). Inadvertent dilation of the pupil may worsen the condition. Here, we present a case of idiopathic panscleritis which clinically masqueraded as an acute attack of primary angle closure glaucoma (ACG).

## Case description

**Figure 1:**
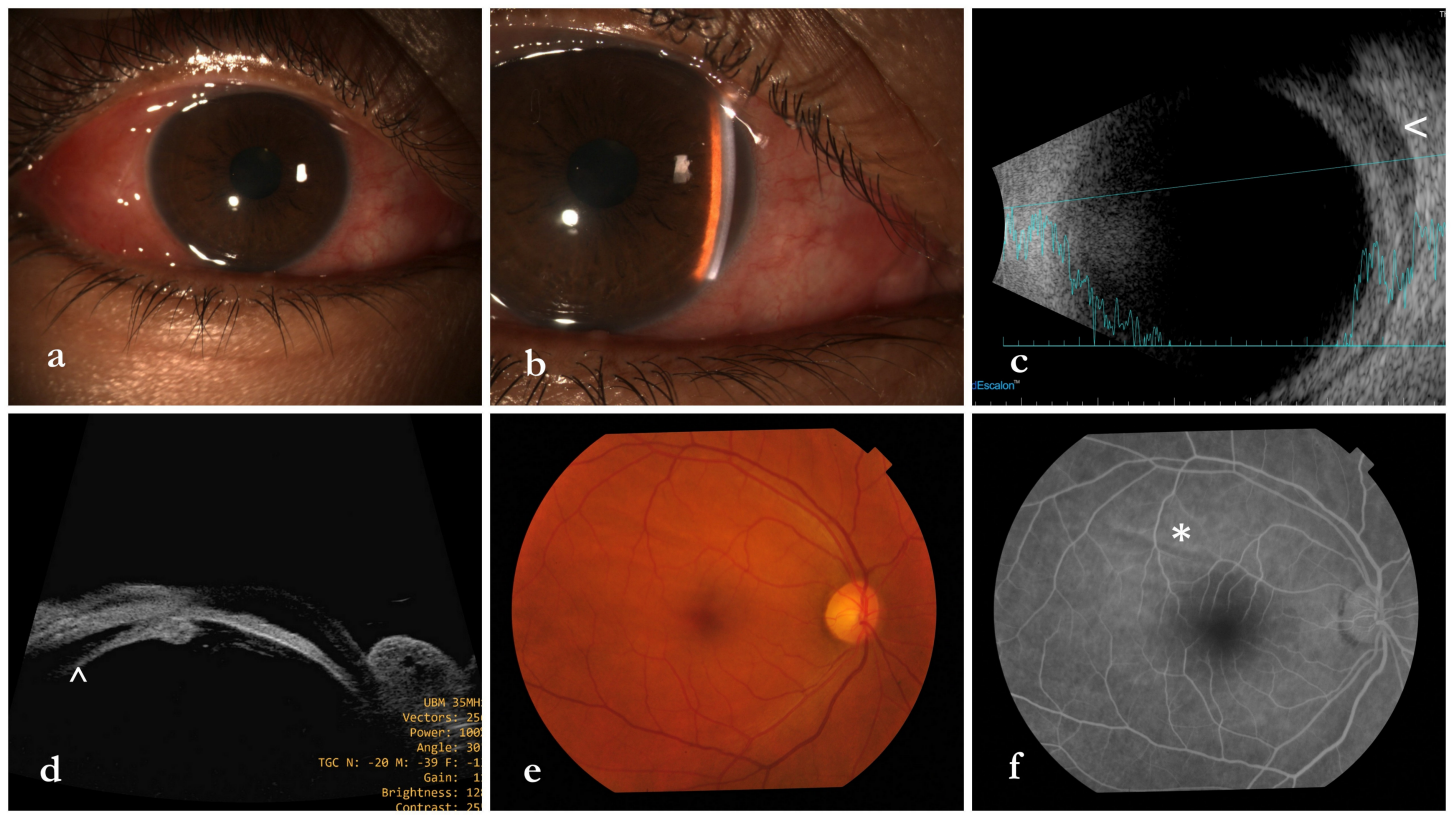
a,b) The slit lamp photos at presentation show the chemosis, conjunctival congestion and shallow anterior chamber with clear cornea. c) The ultrasonogram shows a thick ocular coat with subtenon fluid (<). d) The ultrasound biomicroscopy reveals supraciliary effusion (^) and anterior rotation of the ciliary body. e,f) Fundus photo and fluorescein angiogram show the choroidal folds (*).

**Figure 2:**
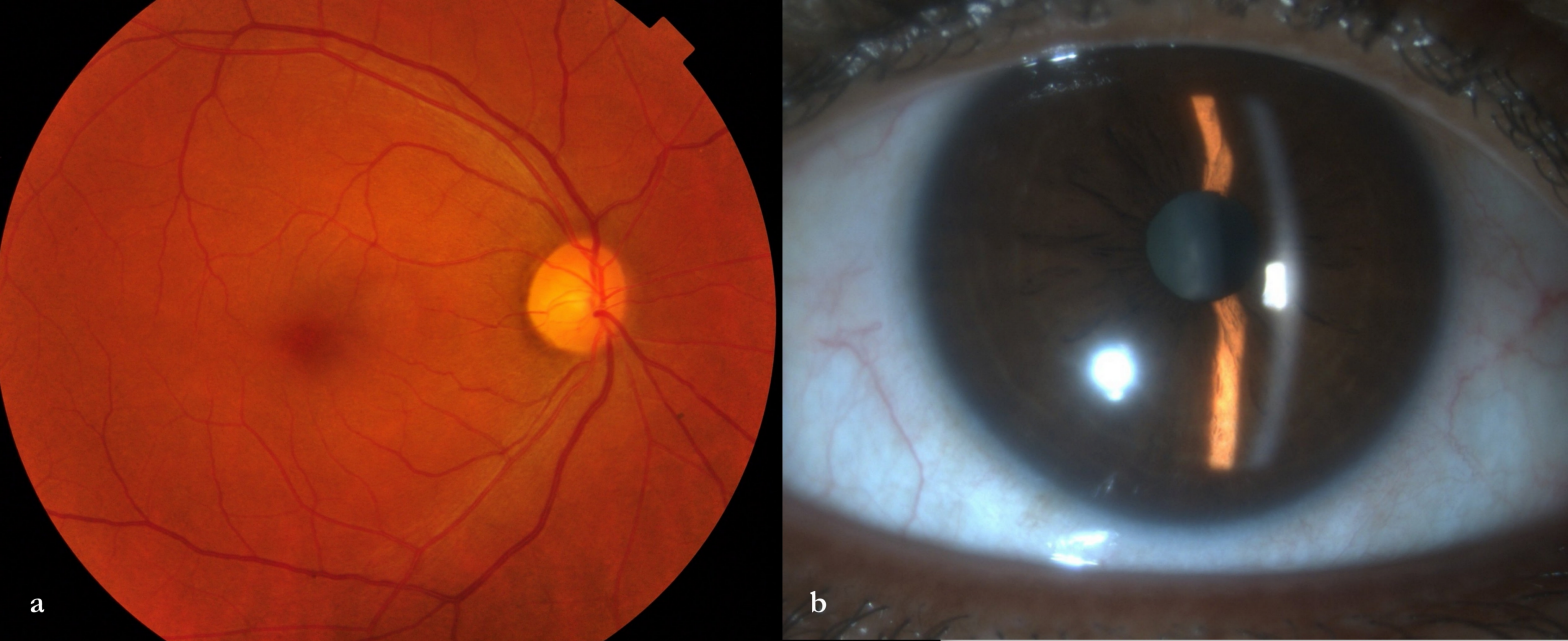
a) The color fundus photo shows resolved choroidal folds 1 month after presentation. b) The anterior chamber had deepened and the eye was quiet at 8-month follow-up.

A 50-year-old female was referred to us as a case of primary acute angle closure attack in the right eye and for laser PI. She had redness, watering, and severe ocular pain in her right eye (RE) for 1 day. The visual acuity was 6/18 in the right eye (RE) and 6/6 in the left eye (LE). LE had a deep anterior chamber (AC) and a normal fundus. Slit lamp examination of RE revealed conjunctival chemosis, circumcorneal congestion, shallow AC, and no AC cells (Figure 1a,b (Fig. 1)). Intraocular pressure (IOP) was 38 mmHg in RE and 12 mmHg in LE by applanation. A diagnosis of acute primary ACG due to pupillary block mechanism in RE was considered. However, additional findings included swelling of the upper eyelid, scleral tenderness, clear cornea, reactive round pupil, fine choroidal folds (Figure 1e (Fig. 1)), shallow peripheral annular choroidal detachment, and limitation of ocular movement with pain in RE. She had a history of such an episode three years back. Due to these factors, a suspicion of scleritis with secondary ACG was also kept as a differential diagnosis. An ultrasonogram (USG) B-scan of RE showed fluid accumulation in the subtenon space with thickening of the ocular coats (Figure 1c (Fig. 1)). Ultrasound biomicroscopy (UBM) revealed thickening of the sclera and supraciliary effusion along with some anterior rotation of the ciliary body (Figure 1d (Fig. 1)). Fundus fluorescein angiography made the choroidal folds obvious (Figure 1f (Fig. 1)). She was diagnosed to have diffuse anterior scleritis and posterior scleritis (panscleritis) with secondary ciliochoroidal effusion and ACG in RE. She was started on topical atropine sulfate drop 1% thrice a day, brimonidine tartrate drop 0.15% twice a day, prednisolone acetate 1% 4 times a day in RE, tablet acetazolamide 250 mg twice daily, and intravenous methylprednisolone 1 mg once daily for three consecutive days. At day two, there was an improvement in lid swelling, ocular pain, ocular motility, conjunctival chemosis, and congestion, and the vision improved to 6/9 in RE. The anterior chamber deepened and the IOP was 8 mmHg in RE. Oral acetazolamide were stopped. After three days of pulse steroid, she was shifted to oral prednisolone 1 mg/kg/day and atropine/prednisolone drops were continued. At 1 week, the UBM showed resolved ciliary effusion. She was negative for antinuclear antibody (ANA), anti-neutrophilic cytoplasmic antibody (P-ANCA, and C-ANCA), anti-citrullinated cyclopeptide (anti-CCP), and VDRL (venereal disease research laboratory). Angiotensin converting enzyme, chest X-ray, and Mantoux were unremarkable. Erythrocyte sedimentation rate was 28 (normal 0–20) mm in the first hour, and C-reactive protein was 21 (normal <10) mg/L. There was no history of tick bite or herpes zoster ophthalmicus. The oral and topical steroid was tapered and atropine/antiglaucoma drops were stopped. At 1 month, her best-corrected visual acuity was 6/6 in either eye, the eyes were quiet, and the choroidal folds in the right eye had disappeared (Figure 2a (Fig. 2)). The eye remained stable till the last follow-up at 8 months, and the anterior chamber remained deep (Figure 2b (Fig. 2)).

## Discussion

Scleritis has been classified into anterior and posterior varieties [1]. Anterior scleritis can be diffuse, nodular, or necrotizing. Both anterior and posterior scleritis may occur simultaneously giving rise to panscleritis [2]. Scleritis is a severe inflammatory condition which may be isolated (idiopathic) or associated with various diseases including rheumatoid arthritis, granulomatosis with polyangiitis (Wegener’s), relapsing polychondritis, infection with tuberculosis, syphilis, and herpes virus. The acute complications of scleritis include disc edema, proptosis, serous retinal detachment, choroidal/corneal involvement, uveitis, vascular occlusions, and glaucoma [1]. Scleritis may be complicated by glaucoma in 11.6% to 49% of cases [1], [3]. Mostly, this angle is open in such cases and causes of IOP rise include damage/inflammation of trabecular meshwork (trabeculitis), increased viscosity of aqueous, occlusion of trabecular meshwork of inflammatory cells, neovascularization of the angle of the anterior chamber, areas of peripheral anterior synechia, steroid response, or increased episcleral venous pressure [4].

However, secondary angle closure glaucoma in scleritis is uncommon but not unknown [4], [5], [6], [7], [8]. It is seen usually following posterior scleritis (non-pupillary block mechanism), but may also be caused by pupillary block [9]. In posterior scleritis, the angle closes and AC becomes shallow due to choroidal and supraciliary effusion with swelling and anterior rotation of the ciliary body which results in a forward shift of the iris lens diaphragm. The same mechanism is seen in ACG in conditions including excessive panretinal photocoagulation and drugs (oral topiramate [10], topical pilocarpine). The management includes cycloplegia with atropine and management of the cause (i.e. opposite to the management of primary acute angle closure glaucoma which includes pilocarpine and PI).

McGavin et al. [9] presented a patient with ‘rheumatoid episcleritis’ with bilateral angle closure glaucoma presumably due to a pupillary block mechanism as the IOP came down after peripheral iridectomy. Quinlan and Hitchings [5] reported 3 cases of posterior scleritis who presented with angle closure glaucoma and peripheral choroidal detachment. Glaucoma resolved on use of oxyphenbutazone and topical mydriatics/cycloplegics. One patient who also had a subretinal (sclera) abscess needed systemic prednisolone for choroidal detachment persisting after 5 days [5]. Phelps presented a case of recurrent scleritis and ciliary body swelling with anterior rotation of ciliary body causing myopia with angle closure glaucoma [11].

Wagemans and Bos [7] reported a 21-year-old female with thrombotic thrombocytopenic purpura and suspected systemic lupus erythematosus who developed angle closure secondary to posterior scleritis with exudative retinal detachment. She had central retinal venous occlusion and the outcome of pars plana vitrectomy with epiretinal membrane peeling and silicone oil for vitreous hemorrhage was dismal with the final visual acuity of light perception [7]. Mangouritsas and Ulbig reported an 81-year-old female who developed unilateral angle closure glaucoma with exudative choroidal detachment in posterior scleritis [8]. Jain et al. [4] presented a 19-year-old male with presumed tubercular posterior scleritis with peripheral annular choroidal detachment and angle closure glaucoma who responded favorably with antitubercular treatment, oral and topical steroid, and cycloplegic. Ugurbas et al. [6] reported a 29-year-old male with proptosis, redness, myopia, and acute angle closure glaucoma who was referred for laser iridotomy. However, the ultrasound confirmed a diagnosis of posterior scleritis with subtenon fluid (T sign) and exudative retinal detachment which resolved after systemic steroid.

Panscleritis can cause secondary acute ACG. Differentiation from primary acute angle closure attack secondary to pupillary block is important as the treatment is opposite – pilocarpine and peripheral iridotomy in primary ACG, atropine and treatment of the cause in secondary ACG due to scleritis. Pilocarpine may worsen ACG secondary to panscleritis as it shifts the iris-lens diaphragm anteriorly. UBM and USG are useful modalities for confirmation of diagnosis.

## Notes

### Competing interests

The authors declare that they have no competing interests.
